# Riluzole prescribing, uptake and treatment discontinuation in people with amyotrophic lateral sclerosis in Scotland

**DOI:** 10.1007/s00415-020-09919-9

**Published:** 2020-05-23

**Authors:** Kiran Jayaprakash, Stella A. Glasmacher, Bernard Pang, Emily Beswick, Arpan R. Mehta, Rachel Dakin, Judith Newton, Siddharthan Chandran, Suvankar Pal, the CARE-MND Consortium, Shuna Colville, Richard Davenport, Ian Morrison, George Gorrie, Callum Duncan, Myles Connor, David Simpson, Ondrej Dolezal, Katja Lassak, Antonella Benvenga, Javier Carod Artal

**Affiliations:** 1 Centre for Clinical Brain Sciences, University of Edinburgh, Chancellor’s Building, 49 Little France Crescent, Edinburgh, EH16 4SB UK; 2 Euan MacDonald Centre for Motor Neurone Disease Research, University of Edinburgh, Edinburgh, UK; 3 Anne Rowling Regenerative Neurology Clinic, Royal Infirmary, Edinburgh, UK; 4 Dementia Research Institute, University of Edinburgh, Edinburgh, UK; 5 Department of Clinical Neurosciences, NHS Lothian, Edinburgh, UK; 6 Department of Neurology, NHS Tayside, Dundee, UK; 7 Institute of Neurosciences, NHS Greater Glasgow and Clyde, Glasgow, UK; 8 Department of Neurology, NHS Grampian, Aberdeen, UK; 9 Department of Neurology, NHS Borders, Melrose, UK; 10 Department of Neurology, NHS Dumfries and Galloway, Dumfries, UK; 11 Department of Neurology, NHS Fife, Kirkcaldy, UK; 12 Department of Neurology, NHS Highland, Inverness, UK

Dear Sirs,

Riluzole is the only globally licensed drug treatment for amyotrophic lateral sclerosis (ALS), a rapidly progressive neurodegenerative condition. Trials and population studies have reported a survival gain of approximately 2–4 months with treatment [1, 2], and a low frequency of adverse effects [3]. The National Institute for Health and Care Excellence (NICE) recommends that clinicians offer riluzole to all people with ALS (pwALS) in the absence of contraindications [4]. However, in practice, prescribing and uptake are likely to be influenced by a number of clinical factors. Current evidence on rate of treatment discontinuation is limited by selection bias, stemming mainly from trials and small observational studies.

We investigated factors influencing riluzole prescription, uptake and discontinuation using data from a large national disease register with 99% case ascertainment.

Participants were drawn from the Clinical Audit Research and Evaluation of MND (CARE-MND) platform, a prospectively maintained population-based register comprising longitudinal clinical, and research data for all pwALS in Scotland [5]. We extracted clinical characteristics of people with definite, probable or possible ALS [6]. Summary statistics are reported as median with interquartile range (IQR). Data were analysed using multivariable multinomial logistic regression and are reported as odds ratio (OR) with 95% confidence intervals (CIs). Missing data were handled using multiple imputation (*m* = 5). Data on the presence/absence of cognitive impairment were used to inform imputation of missing Edinburgh Cognitive and Behavioural Screen (ECAS) scores. Analyses were performed in R (3.6.2.).

768 pwALS were identified between January 2015 and April 2020. 468 pwALS (60.9%) were male, median age at diagnosis was 68 years (IQR 60–75) and median time from onset to diagnosis was 11 months (IQR 7–19). Site of onset was limb (338, 63.3%), bulbar (150, 28.1%), mixed (38, 7.1%) and pure respiratory (8, 1.5%). The median ALSFRS-R score was 38 (IQR 31–42) and the median ECAS score was 109/136 (IQR 91–115, *n* = 253).

Of all pwALS, 632 (86.5%) were offered riluzole and 283 (38.7%) did not commence treatment, which was due to patient preference in 223 (78.8%) cases (Fig. 1). Older age was significantly associated with pwALS not being offered riluzole, and with not starting riluzole. Sex, diagnostic delay, ALSFRS-R total and swallow subscores, and ECAS scores were not associated with prescription or uptake of riluzole (Table 1).

**Fig. 1:**
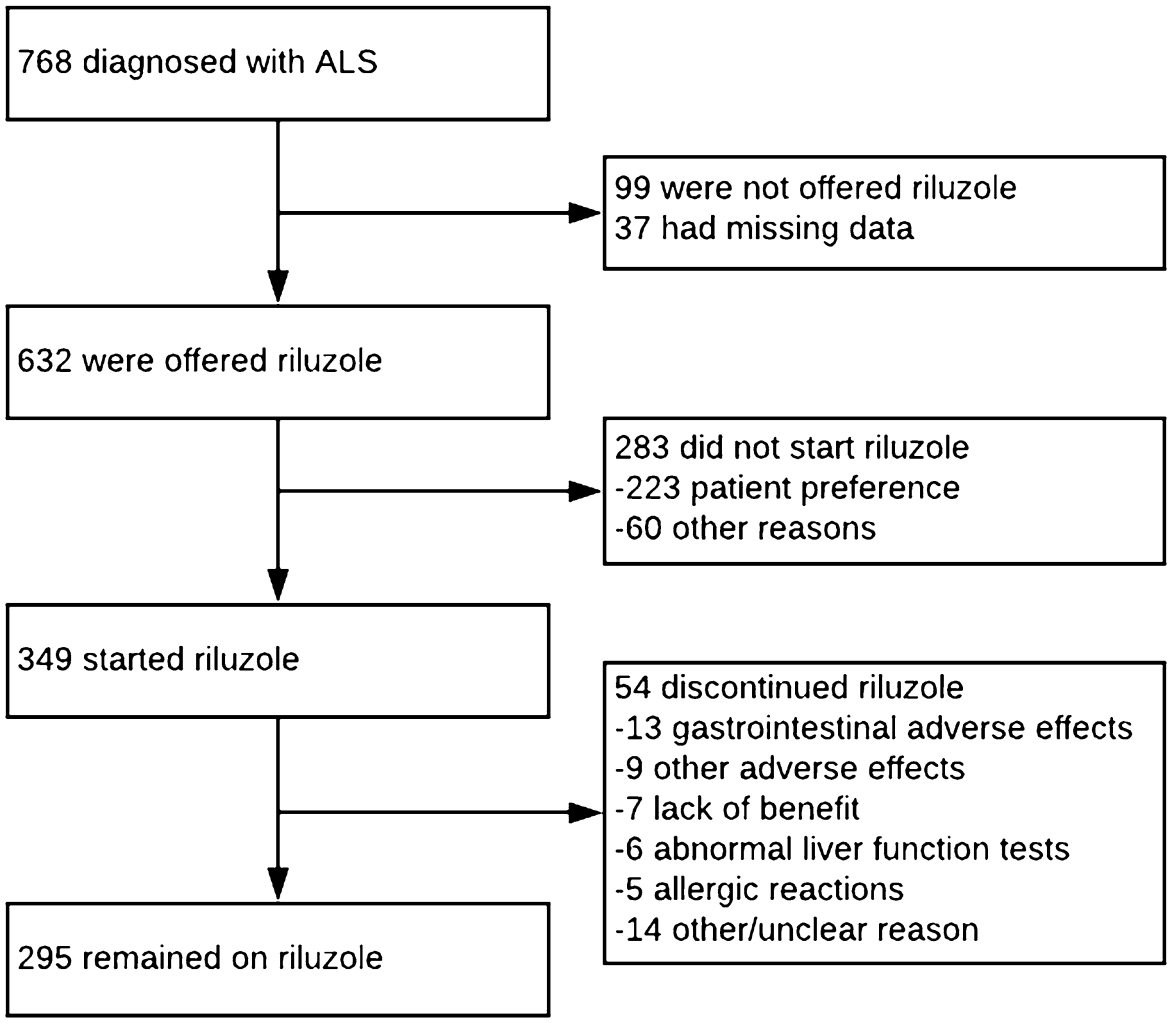
Patient flow chart

**Table 1:** Factors associated with being offered riluzole and not starting riluzole, compared to starting riluzole (reference category)

Characteristic	Not offered riluzole (*n* = 99) OR (95% CIs); *p* value	Offered but not started riluzole (*n* = 283) OR (95% CIs)	Started riluzole (*n* = 349) (Reference category)
Age at clinical ALS diagnosis (years)	1.05 (1.03, 1.08); *p* < 0.001	1.03 (1.01, 1.04); *p* < 0.001	1
Male sex	1.05 (0.65, 1.69); *p* = 0.86	1.07 (0.76, 1.51); *p* = 0.69	1
Time between symptom onset and clinical ALS diagnosis (months)	1.01 (1.00, 1.02); *p* = 0.14	1.00 (0.99, 1.01); *p* = 0.61	1
Total ALSFRS-R score (0–4)	0.97 (0.94, 1.01); *p* = 0.12	0.99 (0.96, 1.01); *p* = 0.36	1
ALSFRS swallow subscore (0–48)	0.77 (0.55, 1.06); *p* = 0.11	0.87 (0.69, 1.10); *p* = 0.24	1
ECAS total score (0–136)	0.99 (0.97, 1.01); *p* = 0.21	0.99 (0.98, 1.00); *p* = 0.08	1

*ALSFRS-R* Amyotrophic lateral sclerosis functional rating scale, *CI* confidence interval, *ECAS* Edinburgh Cognitive and Behavioural Screen, *OR* odds ratio

Of those who started riluzole, 54 (15.4%) subsequently discontinued treatment. The most common reasons for discontinuation were gastrointestinal adverse effects (24.8%), including nausea, abdominal discomfort, constipation, and anorexia. Other adverse effects (including fatigue/malaise) accounted for 13.0%, deranged liver function 11.1%, and allergic reactions 9.3%. 13% discontinued due to ongoing ALS progression. In the remainder, the reason was unclear. Median time until discontinuation was 3 months for adverse effects and ALS progression (IQR 1.8–3.3 and 2.0–5.0, respectively) and 4.5 months for deranged liver function tests (IQR 2.5–6.0).

The proportion of pwALS in Scotland offered riluzole (86.5%) is in keeping with previous estimates of 66–100% in the United Kingdom [7, 8] and 57–85% internationally [9, 10].

Older age was associated with lower rates of riluzole prescription and uptake, which may be because of prescribers’ and/or pwALS' concerns about increased vulnerability to adverse effects owing to comorbidity and polypharmacy. Additionally, therapeutic nihilism regarding the modest survival gain conferred by riluzole may be more prominent in this group; however, recent research found that the survival benefit of riluzole is greater in older pwALS [11]. This study, together with our data, emphasises the importance of offering Riluzole to all pwALS.

15% of pwMND discontinued riluzole; this figure varied between 4–40% in previous research [3]. Our data offer insight into reasons for discontinuation, which may inform pre-treatment discussion with pwALS.

## Data Availability

Upon request from the authors.
